# Supplementary material for: TiO2 (Core)/Crumpled Graphene Oxide (Shell) Nanocomposites Show Enhanced Photodegradation of Carbamazepine
Source: Nanomaterials (Basel). 2021 Aug 17;11(8):2087. doi: 10.3390/nano11082087 (PMC8401461; doi:10.3390/nano11082087)
Supplement: Supplementary file 1 [file nanomaterials-11-02087-s001.zip › nanomaterials-1322242-supplementary.pdf]

nanomaterials

Electronic Supplementary Information

TiO_2 (Core)/Crumpled Graphene Oxide (Shell) Nanocomposites Show Enhanced Photodegradation of Carbamazepine

Han Fu and Kimberly A. Gray *

Department of Civil and Environmental Engineering, Northwestern University, 2145 Sheridan Road, Evanston, IL 60208, USA; hanfu2023@u.northwestern.edu (H.F.)
* Correspondence: k-gray@northwestern.edu (K.G.)

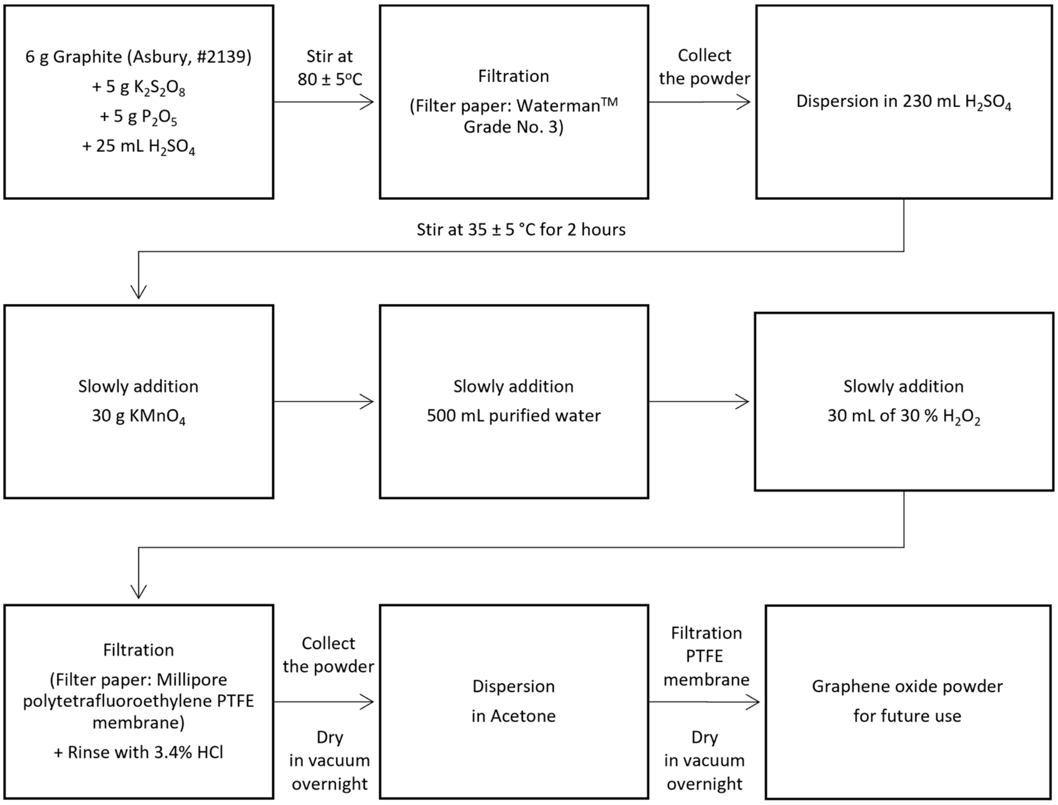

Figure S1. Schematic flow chart of graphene oxide synthesis method. All the chemicals were purchased from Sigma-Aldrich, or Fisher Chemical.

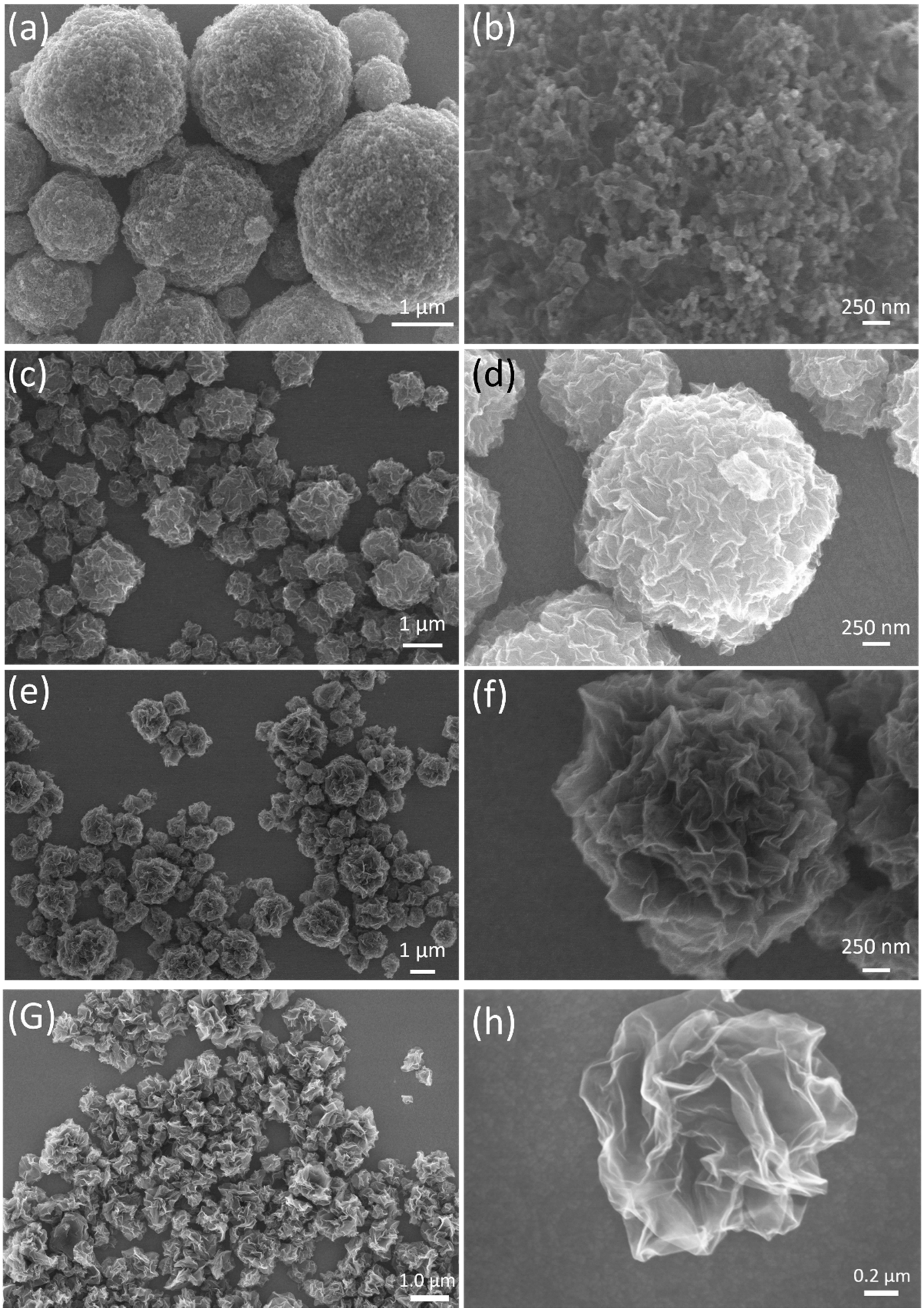

Figure S2. SEM images of TiGC with different TiO_2 and GO weight ratio (**a**,**b**) TiO_2:GO ratio 10:1 (**c**,**d**) TiO_2:GO ratio 5:1 (**e**,**f**) TiO_2:GO ratio 1:1 (**g**,**h**) Crumpled graphene ball (CGB, no presence of TiO_2 during synthesis).

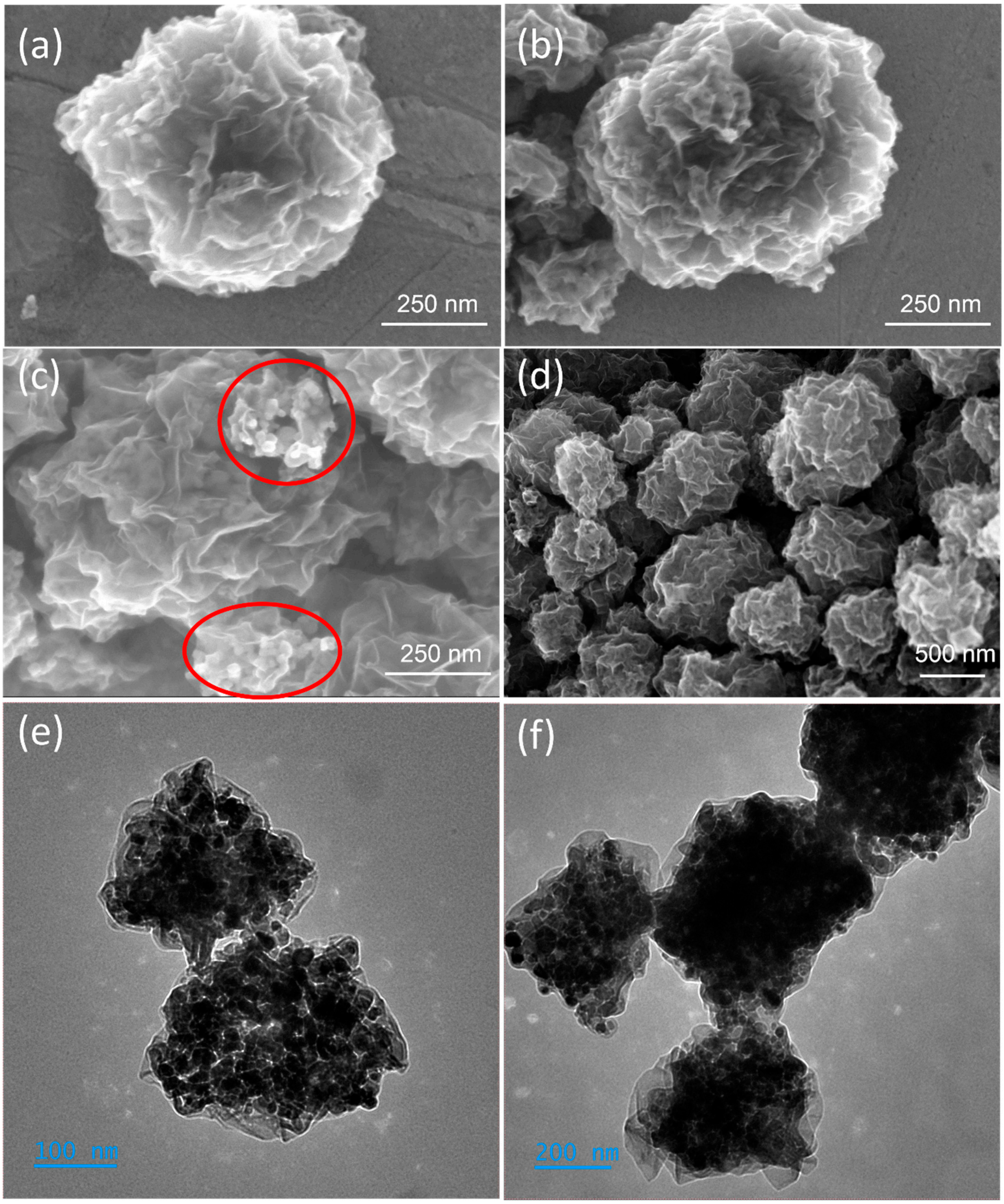

Figure S3. (**a**–**d**) SEM images and (**e**,**f**) TEM images of TiGC (TiO_2:GO weight ratio 2:1) after photodegradation experiment.

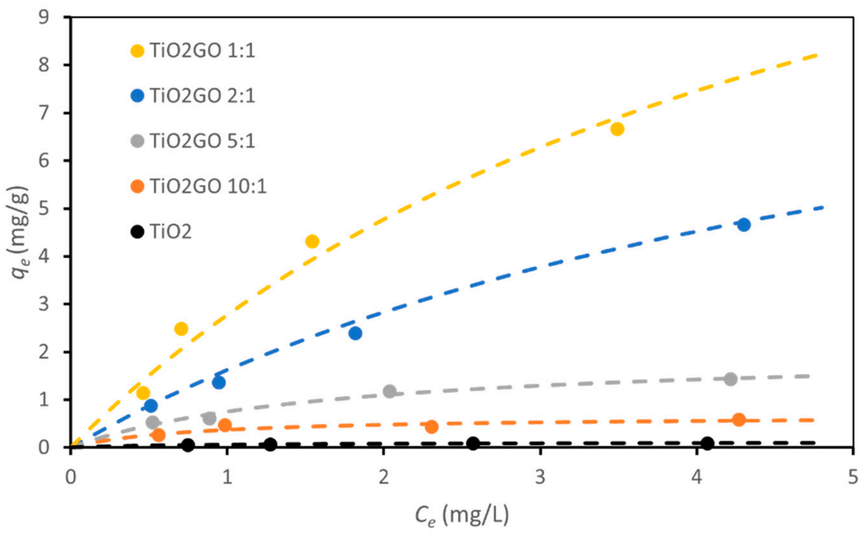

Name	q_m(mg/g)	K_L(L/mg)	R^2
TiGC (TiO_2:GO Ratio 10:1)	1.25	0.67	0.9765
TiGC (TiO_2:GO Ratio 5:1)	2.02	0.59	0.9816
TiGC (TiO_2:GO Ratio 2:1)	11.19	0.17	0.9965
TiGC (TiO_2:GO Ratio 1:1)	17.12	0.19	0.9913
TiO_2	0.12	1.04	0.9936

Figure S4. Langmuir adsorption model for carbamazepine adsorption with different TiO_2 and graphene oxide ratios in TiGC.

Pseudo first-reaction model

Name	k_1 (min^{-1})	R^2
TiGC (TiO_2:GO Ratio 10:1)	0.0269	0.9554
TiGC (TiO_2:GO Ratio 5:1)	0.0326	0.9924
TiGC (TiO_2:GO Ratio 2:1)	0.0286	0.9953
TiGC (TiO_2:GO Ratio 1:1)	0.0177	0.9934
TiO_2	0.0226	0.9914

Langmuir Hinshelwood model

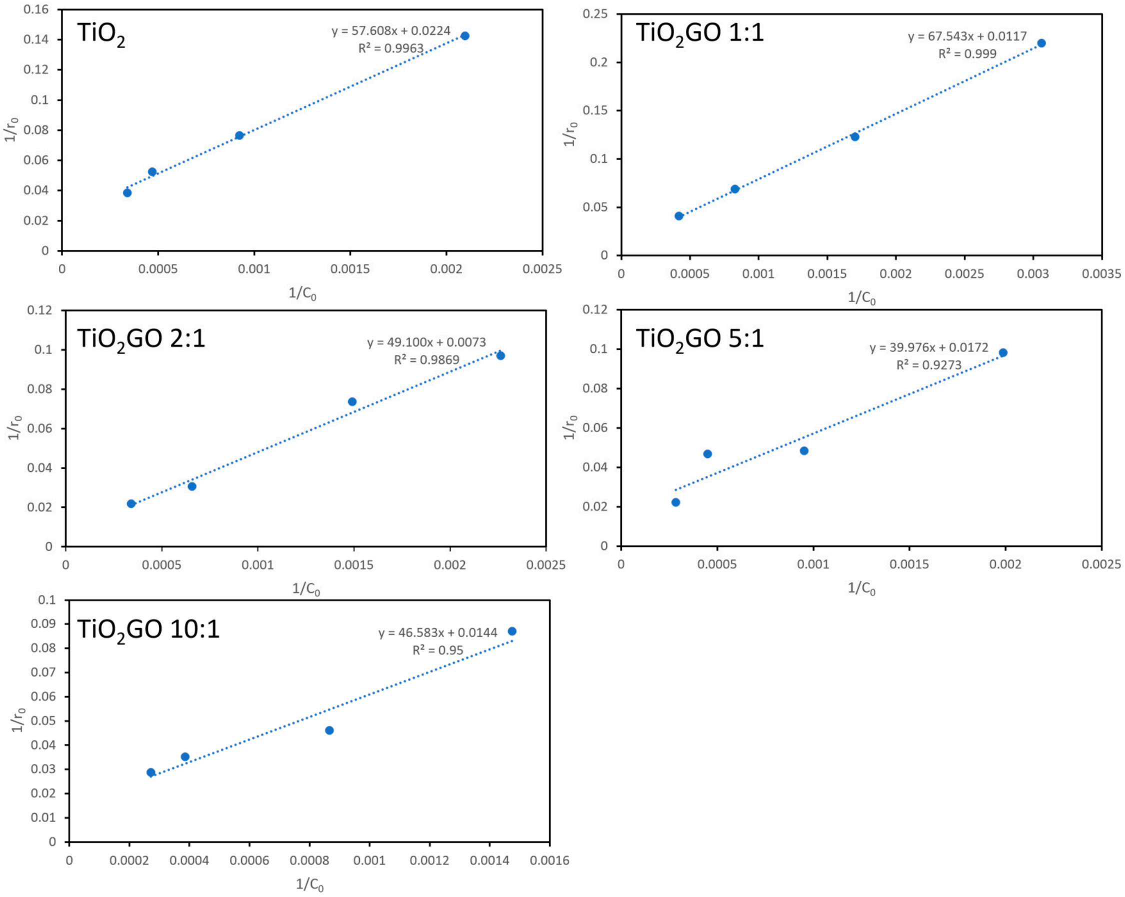

Name	$k_{L\text{-}H}$ (µg L^{-1}min^{-1})	$K_{L\text{-}H}$ (L µg^{-1})	R^2
TiGC (TiO_2:GO Ratio 10:1)	69.45	0.00028	0.9500
TiGC (TiO_2:GO Ratio 5:1)	58.14	0.00043	0.9273
TiGC (TiO_2:GO Ratio 2:1)	142.86	0.00014	0.9869
TiGC (TiO_2:GO Ratio 1:1)	93.46	0.00016	0.9990
TiO_2	51.67	0.00046	0.9963

Figure S5. Pseudo first-reaction model and Langmuir Hinshelwood model calculation for carbamazepine photodegradation with different TiO_2 and graphene oxide ratios in TiGC.

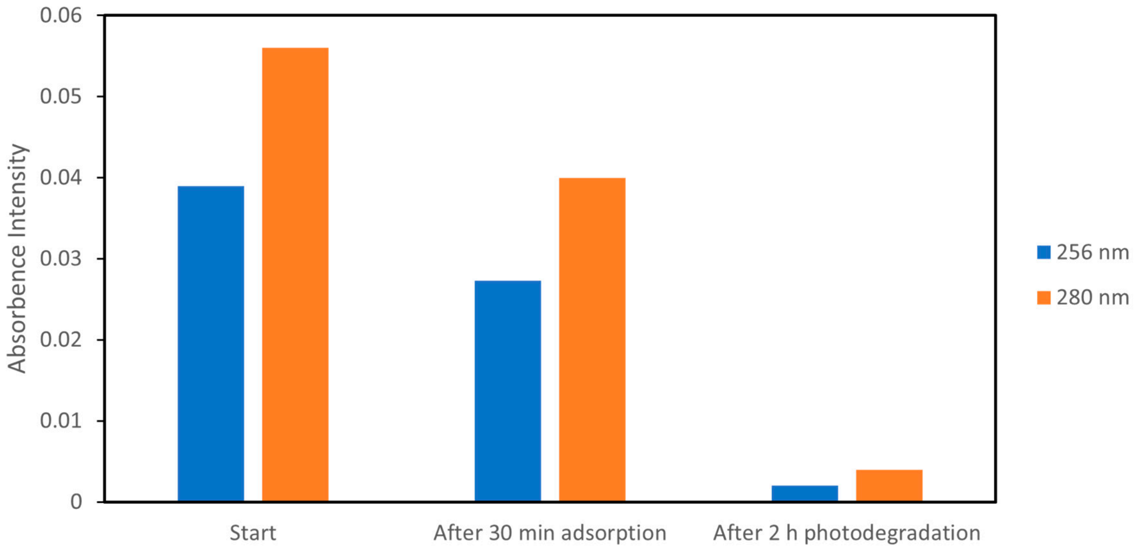

Figure S6. UV-vis spectra of two wavelength 256 nm and 280 nm during the photodegradation experiment.

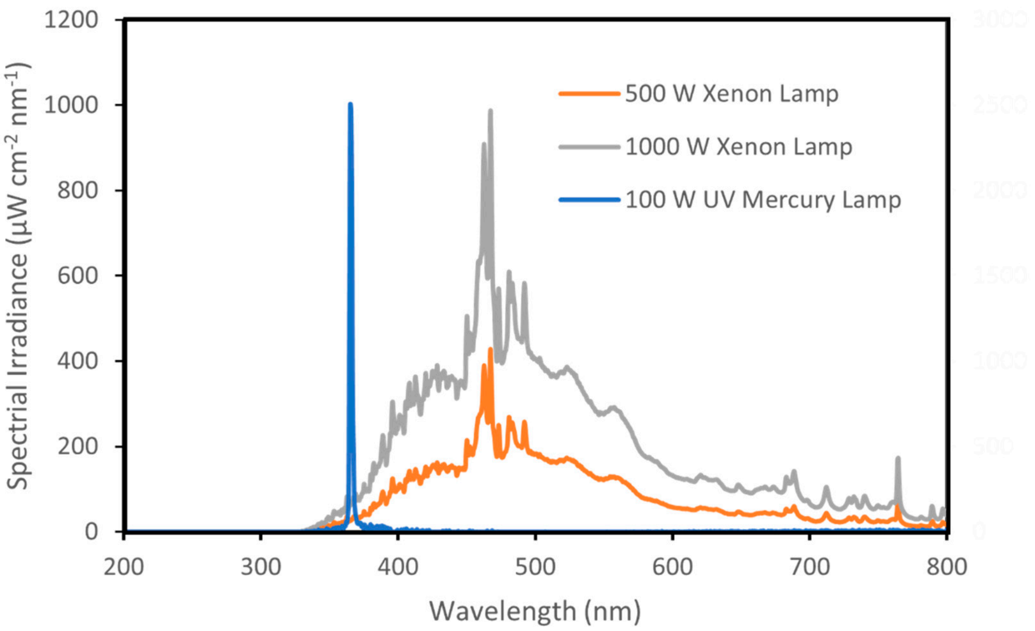

Figure S7. Light spectra of simulated solar irradiation from Mercury Lamp (100 W) Xe arc lamp (500 W and 1000 W) under carbamazepine photodegradation tests.

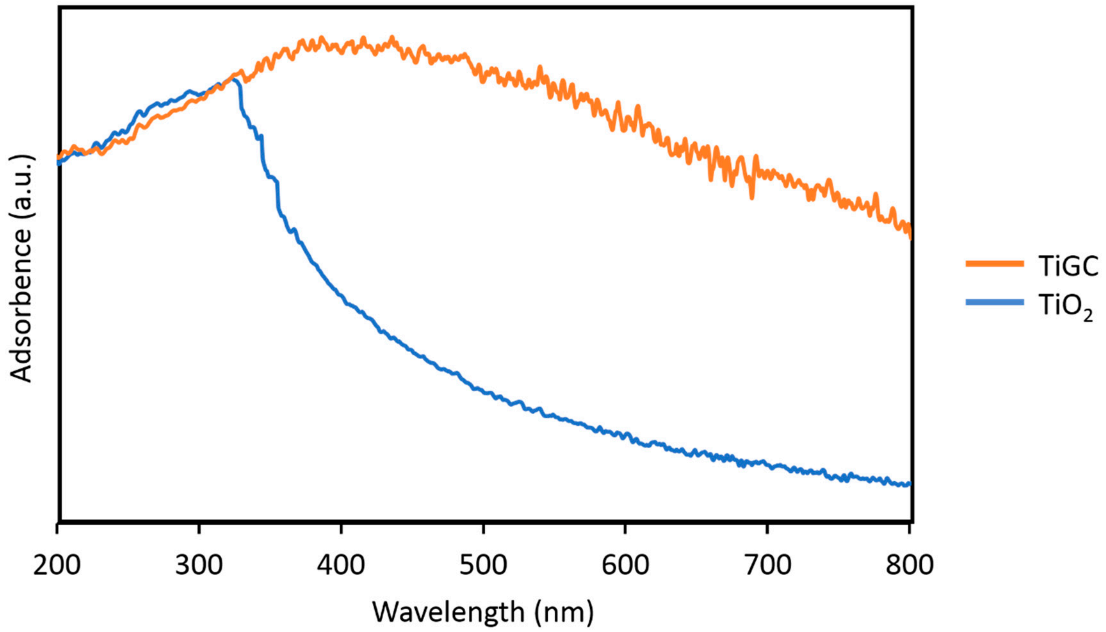

Figure S8. UV-vis spectra of TiO_2 P25 and TiGC (TiO_2:GO ratio 2:1).

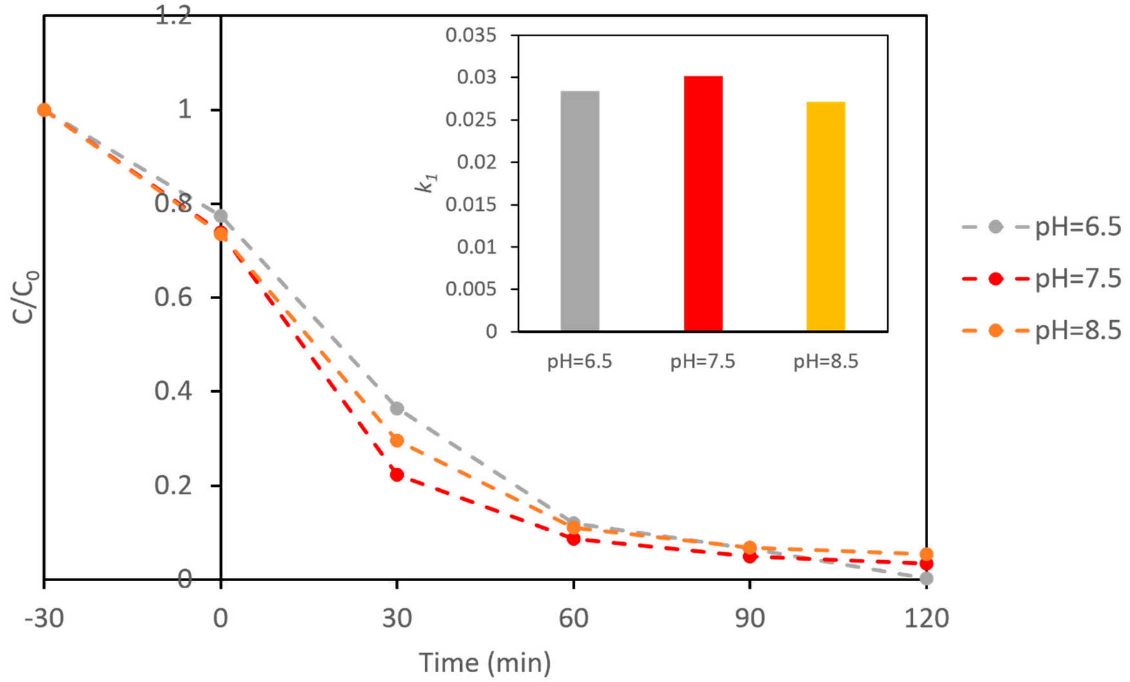

Figure S9. C/C_0 Plot for CBZ photodegradation of TiGC (TiO_2:GO ratio 2:1) and related pseudo first-order reaction constant, k_1 (min^{-1}) at different pH 6.5, 7.5 and 8.5. CBZ initial concentration = 1 mg/L, catalyst dose = 0.2 mg/mL.

Name	q_m(mg/g)	K_L(L/mg)	R^2
Control (purified water)	11.19	0.17	0.9913
NOM 10 mg/L	11.23	0.16	0.9969
NOM 20 mg/L	12.41	0.13	0.9915

Figure S10. Langmuir adsorption model for carbamazepine adsorption with TiGC (TiO_2:GO ratio 2:1) under different NOM concentrations.
